# COP1 controls salt stress tolerance by modulating sucrose content

**DOI:** 10.1080/15592324.2022.2096784

**Published:** 2022-07-14

**Authors:** Joo Yong Kim, Seung Ju Lee, Wang Ki Min, Seoyeon Cha, Jong Tae Song, Hak Soo Seo

**Affiliations:** a Department of Agriculture, Forestry and Bioresources, Research Institute of Agriculture and Life Sciences, Seoul National University, Seoul, Korea; b Department of Applied Biosciences, Kyungpook National University, Daegu, Korea; c Institute, Seoul National UniversityBio-MAX, Seoul, Korea

**Keywords:** Arabidopsis, COP1, *cop1-4* mutant, *cop1-6* mutant, root growth, salt tolerance, sucrose

## Abstract

The E3 ubiquitin ligase Constitutive Photomorphogenic 1 (COP1) plays evolutionarily conserved and divergent roles. In plants, COP1 regulates a large number of developmental processes including photomorphogenesis, seedling emergence, and gravitropism. Nevertheless, its function in abiotic stress tolerance remains largely unknown. Here, we demonstrate the role of COP1 in salt stress tolerance in *Arabidopsis thaliana*. In soil, *cop1-4* and *cop1-6* mutants were more tolerant to high salinity than wild-type (WT) plants during vegetative growth. However, in high salt-containing Murashige and Skoog (MS) medium, *cop1-4* and *cop1-6* seedlings exhibited significantly impaired growth compared with WT plants. Notably, *cop1-4* and *cop1-6* seedlings recovered their growth to the WT level upon exogenous sucrose treatment even under high salinity conditions. Compared with WT plants, the sucrose content of *cop1-4* mutants was much higher at the vegetative growth stage but similar at the seedling stage. Upon exogenous sucrose supply, root elongation was significantly stimulated in *cop1-4* seedlings but only slightly stimulated in WT plants. Thus, no significant difference was observed in root length between the two genotypes. Altogether, our data indicate that *cop1* mutants are more tolerant to salt stress than WT plants, and the salt tolerance of *cop1* mutants is correlated with their sucrose content.

## Results and discussion

COP1 is a major controller of light-responsive genes, and exerts its function by destabilizing various transcriptional regulators including Long After Far-red light 1 (LAF1) and Elongated Hypocotyl 5 (HY5), which are involved in photomorphogenesis.^1–3^ In the plant kingdom, besides photomorphogenesis, COP1 plays crucial roles in various developmental processes such as photoprotection in algae and gravitropism in mosses.^4–8^ In mammals, COP1 has been reported to participate in metabolism,^9,10^ tumorigenesis,^11–15^ and neuron development.^16^ Moreover, COP1 is also involved in the response to abiotic stresses, such as cold^17^ and UV-B exposure,^18^ and biotic stresses, such as viral infection.^19,20^ In Arabidopsis, it has been recently demonstrated that the E3 ubiquitin ligase activity of COP1 is involved in abiotic stress response via the modulation of the E3 SUMO ligase AtSIZ1 level.^21^ However, the direct involvement of COP1 in the response to salt stress has not yet been elucidated in plants. Therefore, in this study, we investigated the role of COP1 in salt stress response.

**Figure 1.**
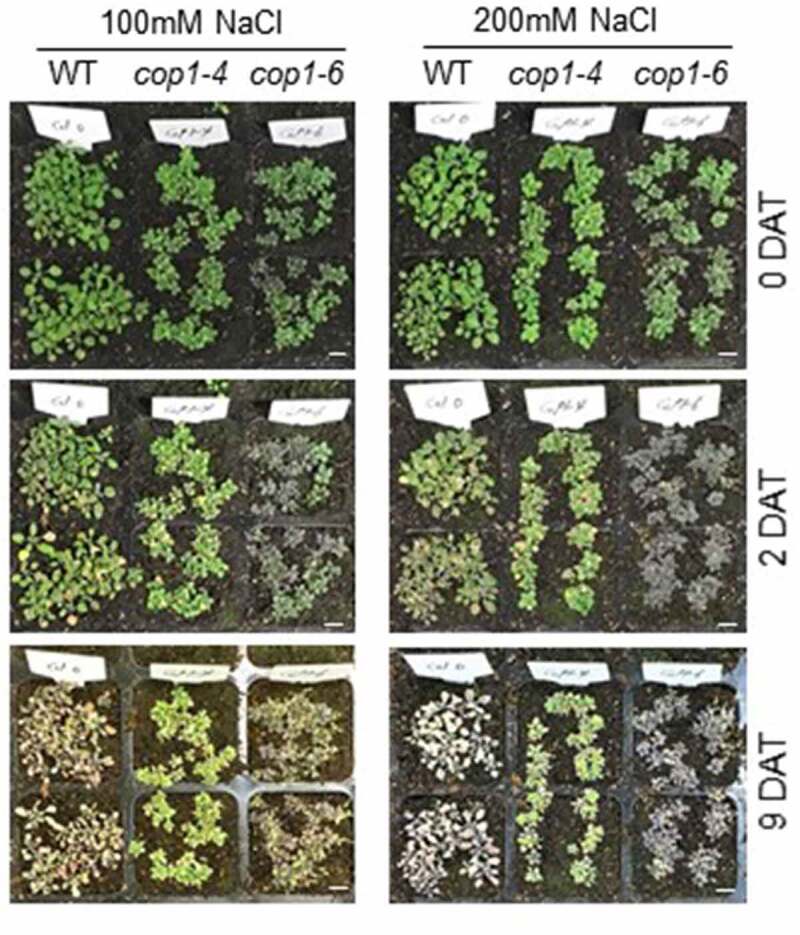
Phenotypic evaluation of the salt tolerance of *cop1-4* and *cop1-6* mutants. Seeds of WT, *cop1-4*, and *cop1-6* mutants were directly sown in soil. After 16 days of growth, the plants were treated with 100 or 200 mM NaCl for 9 days and then photographed. DAT, days after treatment. Scale bars = 1 cm.

**Figure 2.**
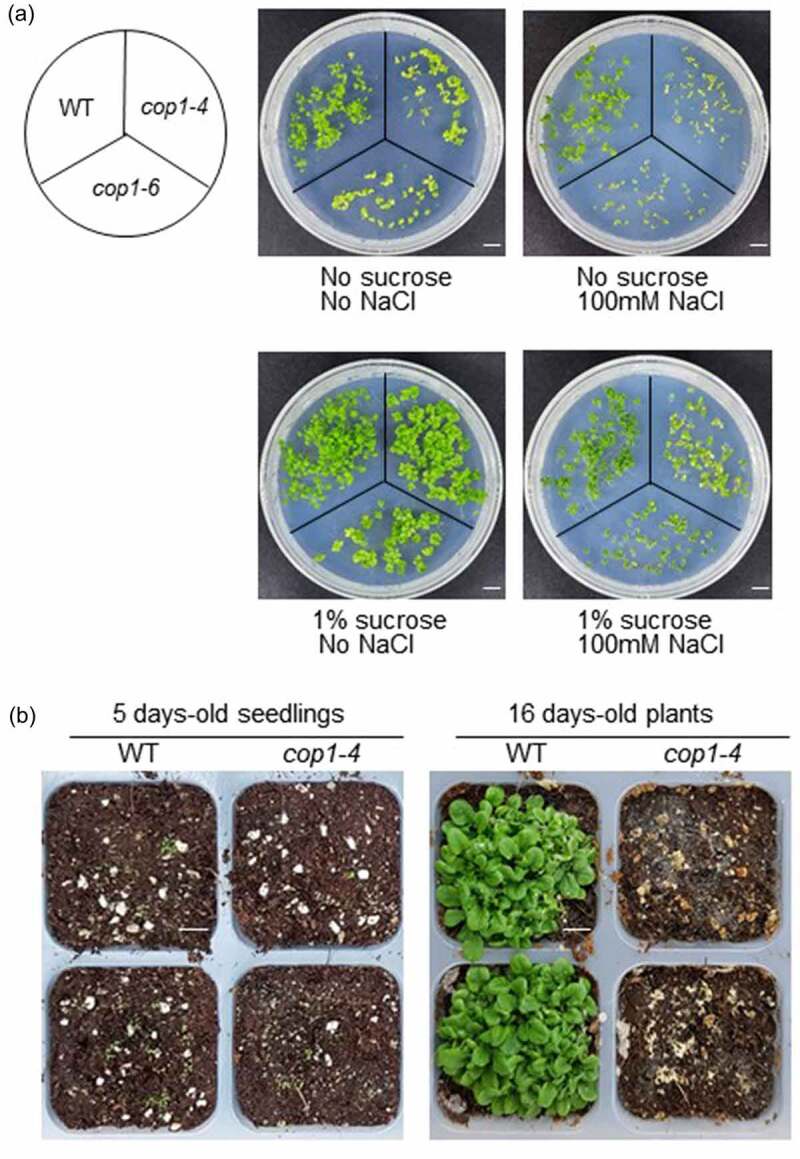
Effect of sucrose on the salt stress tolerance of *cop1-4* and *cop1-6* mutants. (a) Seeds of WT, *cop1-4* and *cop1-6* mutants were germinated on salt (100 mM NaCl)-containing MS media supplemented with or without 1% sucrose. Plants were photographed after 7 days. Scale bars = 1 cm. (b) Seeds of WT and *cop1-4* mutants were sown directly in soil. After 5 days, seedlings were treated with 100 mM NaCl. Plants were photographed 11 days later. Scale bars = 1 cm.

Several years ago, it was reported that *cop1-4* mutants are more tolerant to drought stress than the WT.^22^ Therefore, we extended our study to examine the role of COP1 under the high salinity condition. WT, *cop1-4*, and *cop1-6* mutant seeds were germinated in soil. After 16 days of growth, the plants were treated with 100 or 200 mM NaCl, and their salt tolerance was phenotypically evaluated after 9 days. The results showed that *cop1-4* and *cop1-6* mutants were more tolerant to salt stress than WT plants under both 100 and 200 mM NaCl conditions (Figure 1). After 9 days under the 200 mM NaCl condition, WT plants died while *cop1-4* and *cop1-6* mutants remained alive. Interestingly, the leaf color of the *cop1-6* mutant changed from green to rosy brown under salt stress, suggesting that it had accumulated specific metabolites under the high salt condition. Next, we examined the salt tolerance of *cop1-4* and *cop1-6* mutants at the seedling stage. In this experiment, the WT, *cop1-4*, and *cop1-6* seeds were sown on MS media containing 100 mM NaCl, and plant growth was monitored. We found that *cop1-4* and *cop1-6* mutants showed greater growth retardation than WT plants (Figure 2a, upper panel). In a previous study, *cop1-4* seeds were more sensitive to NaCl than WT seeds during germination.^23^ Thus, results of the previous study and current study strongly indicate that *cop1* mutants are more sensitive to salt stress than the WT at the germination and seedling stages.

Exogenous sucrose treatment has been shown to enhance the salt stress tolerance of Arabidopsis seedlings.^24^ To verify whether this result applies to *cop1-4* and *cop1-6* mutants, we added sucrose to salt (100 mM NaCl)-containing MS media and monitored the growth of *cop1-4* and *cop1-6* mutants. Interestingly, the growth of *cop1-4* and *cop1-6* mutants recovered to the WT level in media containing 1% sucrose (Figure 2a, lower panel). Next, to examine the salt tolerance of soil-grown *cop1-4* and *cop1-6* mutants at the seedling stage, WT and *cop1-4* seeds were sown in soil. After 5 days, 100 mM NaCl was added directly to the soil, and seedlings were grown further for 16 days. While the WT plants showed normal growth, *cop1-4* mutant seedlings failed to grow and eventually died (Figure 2b), indicating that *cop1-4* mutants are very sensitive to salt stress at the seedling stage.

**Figure 3.**
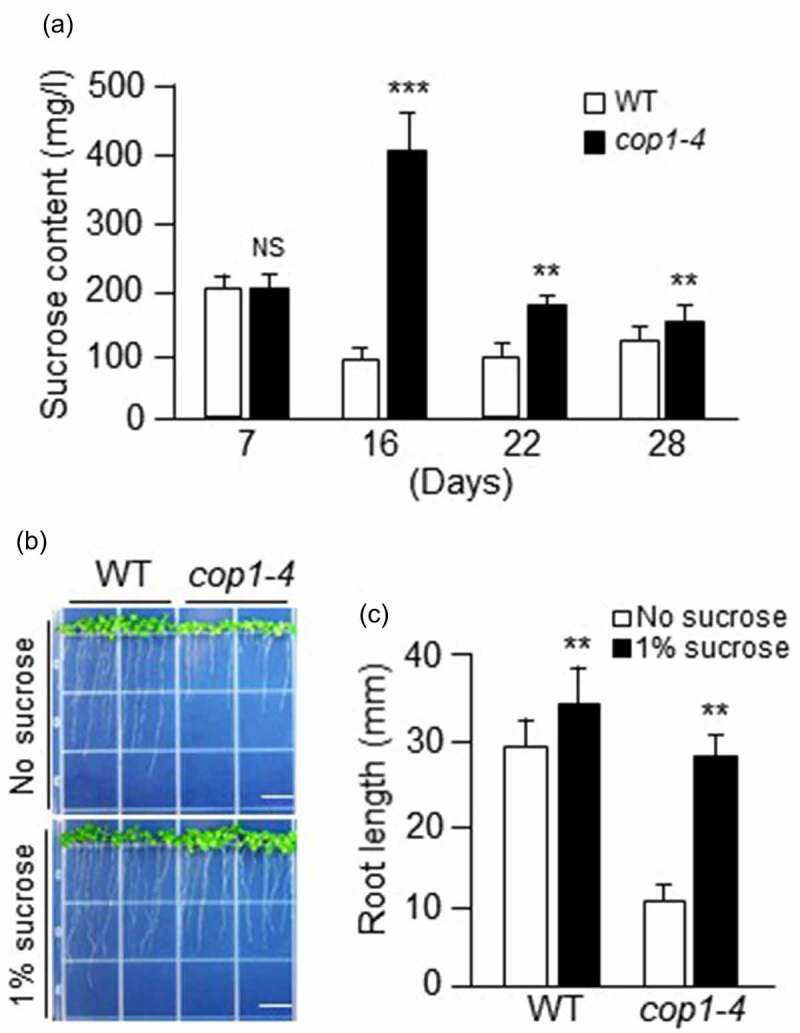
Evaluation of the sucrose content of *cop1-4* mutants and the effect of sucrose on root growth. (a) WT and *cop1-4* mutants were grown in soil for 4 weeks, and sucrose contents were measured using leaf samples harvested at the indicated time points. The leaves of each sample were frozen immediately and ground. The powder was extracted with extraction buffer 1 (EB1) (100 mM HEPES-KOH (pH 7.7) containing 80 ml EtOH 100% (v/v)). The ratio of powered tissue to EB1 was 1:1 (w/v). Samples were heated at 80°C for 2 hours. After centrifugation at 13,000 rpm for 10 minutes, the supernatants were evaporated in a vacuum at 45°C for 2 hours. The dried samples were resuspended with ddH_2_O, filtered through PVDF filters (pore size, 0.45 μm) and then analyzed on a HPLC system. Data are mean values ± SD (Standard deviation). Asterisks indicate significant differences between WT and *cop1-4* (Student’s t-test, **P < .01 and ***P < .001). NS, not significant. (b) WT and *cop1-4* mutants were grown for 10 days on MS media supplemented with or without 1% sucrose. Scale bars = 1 cm. (c) Root length of WT and *cop1-4* mutants. Measurements were taken using samples shown in panel (B). Data are mean values ± SD. Asterisks indicate significant differences between WT and *cop1-4* (Student’s t-test, **P < .01) .

Because *cop1* mutants were sensitive to salt stress at the seedling stage but tolerant to salt stress at the vegetative growth stage, and could recover their growth following exogenous sucrose treatment at the seedling stage, we measured their sucrose content both at the seedling and vegetative growth stages. Sucrose was extracted from the leaf samples of WT and *cop1-4* mutant plants at different growth stages, and quantified (Figure 3a). The amount of sucrose in 7-day-old WT and *cop1-4* seedlings was similar; however, the sucrose contents of 16-day-old *cop1-4* and WT plants were approximately 2-fold higher and lower, respectively, than that of their 7-day-old counterparts. At 16 days, the sucrose content of *cop1-4* mutants was 4-fold higher than that of WT plants. Although the sucrose content of *cop1-4* mutants decreased considerably at 22 days, it was still 2-fold higher than that of WT plants. At 22 days, the level of sucrose in *cop1-4* mutants was slightly higher than that in WT plants. These data indicate that the salt tolerance of *cop1-4* mutants during vegetative growth is derived from their high sucrose content.

Since *cop1* mutant seedlings could recover from salt stress following exogenous sucrose supply, we investigated the sucrose sensitivity of WT and *cop1-4* mutant plants by measuring root growth. WT and *cop1-4* seeds were germinated on MS media supplemented with or without 1% sucrose, and the root length of seedlings was measured after 10 days. On MS media lacking sucrose, the roots of *cop1-4* mutants were 3-fold shorter than those of WT plants (Figure 3b,c). When treated with exogenous sucrose, the growth of *cop1-4* mutant roots was significantly stimulated, whereas that of WT roots was only slightly stimulated (Figure 3b,c). The root length of *cop1-4* mutant plants increased by approximately 3-fold upon exogenous sucrose supply (Figure 3b,c). In sucrose-containing media, the roots of *cop1-4* seedlings were slightly shorter than those of WT seedlings (Figure 3b,c), which explains why *cop1-4* mutant seedlings could recover from salt stress upon exogenous sucrose supply.

**Figure 4.**
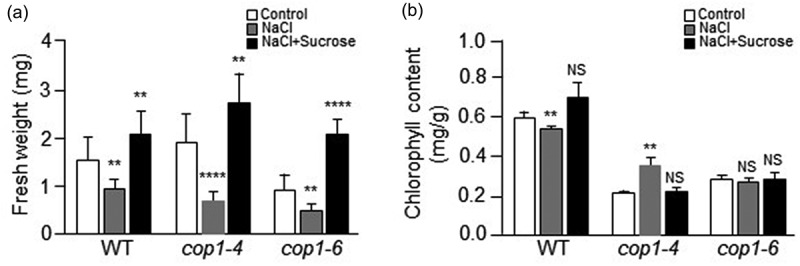
Effect of salt stress on fresh weight and chlorophyll content of *cop1-4* and *cop1-6* mutants. (a) Seeds of WT, *cop1-4*, and *cop1-6* mutants were germinated for 7 days in MS media containing 1% sucrose, 100 mM NaCl, or both 1% sucrose and 100 mM NaCl. The average fresh weight was estimated in three independent experiments using five plants in each experiment. Each bar indicates the average fresh weight of the plants. Data are mean values ± SD. Asterisks indicate significant differences between control and NaCl-containing MS media or between control and both NaCl and sucrose-containing MS media (Student’s t-test, **P < .01, ****P < .0001). (b) Seeds of WT, *cop1-4*, and *cop1-6* mutants were germinated for 7 days on MS media containing 1% sucrose, 100 mM NaCl, or both 1% sucrose and 100 mM NaCl. For measurement of chlorophyll content, total chlorophyll was extracted from the leaves of WT, *cop1-4*, and *cop1-6* mutants with 80% acetone and centrifuged at 13,000 × g for 15 minutes. Absorbance of the supernatants was read at 645 and 663 nm in a UV-spectrophotometer. Total chlorophyll content was calculated using the following equation: Total chlorophyll (mg/g) = (20.2 × A645 + 8.02 × A663) × V/(W × 1000), where V denotes extract volume (ml), and W denotes weight (g) of leaves. Data are mean values ± SD. Asterisks indicate significant differences between control and NaCl-containing MS media or between control MS and both NaCl and sucrose-containing MS media (Student’s t-test, **P < .01). NS, Not significant.

We also examined the effect of salt stress on the fresh weight and chlorophyll content of *cop1* mutants. We first measured the fresh weight of 7-day-old WT, *cop1-4*, and *cop1-6* seedlings (Figure 4a). In control MS media, the fresh weight of the *cop1-4* mutant plant was slightly higher than that of the WT plant, while the fresh weight of the *cop1-6* mutant was slightly lower than that of WT plant. In NaCl-containing media, the fresh weights of WT, *cop1-4*, and *cop1-6* plants were 2-fold lower than under the control condition. However, in NaCl-containing media, the fresh weight of the WT was higher than those of *cop1-4* and *cop1-6* mutants. These results indicate that salt stress impeded more the growth of *cop1-4* and *cop1-6* than that of the WT, which is consistent with the results shown in Figure 2a. In NaCl-containing media supplemented with sucrose, the fresh weights of WT and *cop1-4* plants were slightly higher than under the control condition, whereas the fresh weight of the *cop1-6* mutant was 2-fold higher. These results indicate that salt-stress induced growth retardation of *cop1-4* and *cop1-6* plants was overcome by the addition of sucrose to the growth medium, which is consistent with the results shown in Figure 2a. Therefore, the data indicate that sucrose was required for the growth recovery of *cop1-4* and *cop1-6* mutants under salt stress.

Next, we analyzed the chlorophyll contents of 7-day-old WT, *cop1-4*, and *cop1-6* seedlings (Figure 4b). In control MS media, the chlorophyll contents of *cop1-4* and *cop1-6* mutants were about 3- to 2-fold lower than that of the WT plant, respectively. In NaCl-containing media, the chlorophyll content of the WT was slightly lower than under the control condition, whereas the chlorophyll contents of the *cop1-4* mutants and *cop1-6* mutants were approximately 1.7-fold higher and unchanged, respectively. In NaCl-containing media supplemented with sucrose, the chlorophyll content of the WT was slightly higher than under the control condition, whereas the chlorophyll contents of *cop1-4* and *cop1-6* mutants were unchanged. A previous study reported that chloroplasts of NaCl-grown 14-day-old Arabidopsis plants were impaired, with less developed thylakoid and granum membranes than those of control seedlings.^25^ In addition, the chlorophyll content of NaCl-grown plants is much lower than that of plants grown in the absence of NaCl.^25^ Unexpectedly, despite the growth retardation of 7-day-old *cop1-4* and *cop1-6* seedlings under the salt stress condition (Figure 2a), the chlorophyll contents of *cop1-4* and *cop1-6* mutants in NaCl-containing MS media were not lower than those in control MS media (Figure 4b). In the presence of NaCl, the chlorophyll content of the *cop1-4* mutant was increased, whereas that of the *cop1-6* mutant was not significantly different (Figure 4b). It has also been reported that treatment of Arabidopsis plants with sucrose increases chlorophyll content.^24^ Therefore, on the bases of the previous study and our current data, we speculate that there may be some difference in chlorophyll metabolism between 7-day-old seedlings and 14-day-old plants subjected to salt stress, in particular between 7-day-old *cop1* mutant seedlings and 14-day-old *cop1* mutant plants.

Finally, we estimated the effect of the salt on the sucrose content of *cop1* mutants. Seeds of WT and *cop1-4* plants were germinated in soil. After 10 days, plants were treated with 50 or 200 mM NaCl, and further grown for 5 days. Sucrose contents were measured using leaf samples of the plants as shown in Figure 3a. Under the non-treated condition, sucrose content was approximately 2-fold higher in the *cop1-4* mutant than in the WT (Table 1). Under the 50 mM NaCl condition, the sucrose content of the WT was slightly higher than under the non-treated condition, whereas it was unchanged in the *cop1-4* mutant (Table 1). Under the 200 mM condition, sucrose contents of WT and *cop1-4* plants were approximately 2-fold higher than under the non-treated condition (Table 1). However, it was still 2-fold higher in the *cop1-4* mutant than in the WT plant (Table 1). Again, these data strongly imply that sucrose synthesis-related metabolism is upregulated in *cop1-4* mutants at the vegetative growth stage and that salt stress tolerance of the *cop1-4* mutant at the vegetative stage is enhanced by high sucrose content.

**Table 1.** The effect of salt stress on the sucrose content of *cop1-4* mutants.

	Sucrose content (mg/I)	
NaCI (mM)	WT	*Cop 1–4*
0	372.11	661.85
50	431.06	657.09
200	614.59	1119.74

In conclusion, *cop1* mutants were more tolerant of salt stress than WT plants at the vegetative growth stage. However, at the seedling stage, *cop1* mutants were more sensitive to salt stress than WT plants and their fresh weight was lower than that of the WT under salt stress, although the sucrose content of both genotypes was similar (Figure 2a and Figure 3a). Interestingly, *cop1* mutant seedlings recovered from salt stress upon exogenous sucrose supply. Currently, we do not know why *cop1* mutants are more sensitive to salt stress than the WT at the seedling stage. It is well known that developmental age is a strong determinant of the stress response in plants.^26^ Thus, we tentatively suggest that the sucrose-mediated salt stress response is downregulated in *cop1* mutants at the seedling stage. The level of sucrose was much higher in *cop1* mutants than in the WT at the vegetative growth stage (Figure 3a, Table 1). Studies have shown that sucrose acts as a signaling molecule and is involved in the response to a number of stresses.^27–29^ In Arabidopsis, the loss of sucrose transporters AtSUC2 and AtSUC4 affects sucrose distribution in plant shoots and roots, resulting in hypersensitivity to abiotic stresses and exogenous abscisic acid (ABA) treatment during seed germination and seedling growth.^30^ Thus, all of our data indicate that COP1-mediated salt stress tolerance is correlated with plant sucrose content.

## References

[1] Osterlund MT, Hardtke CS, Wei N, Deng XW. Targeted destabilization of HY5 during light-regulated development of Arabidopsis. Nature. 2000;405:462–6. doi:10.1038/35013076. 10839542

[2] Seo HS, Yang JY, Ishikawa M, Bolle C, Ballesteros ML, Chua NH. LAF1 ubiquitination by COP1 controls photomorphogenesis and is stimulated by SPA1. Nature. 2003;423:995–999. doi:10.1038/nature01696. 12827204

[3] Kim JY, Song JT, Seo HS. COP1 regulates plant growth and development in response to light at the post-translational level. J Exp Bot. 2017;68(17):4737–4748. doi:10.1093/jxb/erx312. 28992300

[4] Schierenbeck L, Ries D, Rogge K, Grewe S, Weisshaar B, Kruse O. Fast forward genetics to identify mutations causing a high light tolerant phenotype in *Chlamydomonas reinhardtii* by whole-genome-sequencing. BMC Genomics. 2015;16:57. doi:10.1186/s12864-015-1232-y. 25730202 PMC4336690

[5] Tilbrook K, Dubois M, Crocco CD, Yin R, Chappuis R, Allorent G, Schmid-Siegert E, Goldschmidt-Clermont M, Ulm R. UV-B perception and acclimation in *Chlamydomonas reinhardtii*. Plant Cell. 2016;28:966–983. doi:10.1105/tpc.15.00287. 27020958 PMC4863380

[6] Artz O, Dickopf S, Ranjan A, Kreiss M, Abraham ET, Boll V, Rensing SA, Hoecker U. Characterization of spa mutants in the moss Physcomitrella provides evidence for functional divergence of SPA genes during the evolution of land plants. New Phytol. 2019;224:1412–14134. 31222750 10.1111/nph.16004

[7] Gabilly ST, Baker CR, Wakao S, Crisanto T, Guan K, Bi K, Guiet E, Guadagno CR, Niyogi KK. Regulation of photoprotection gene expression in Chlamydomonas by a putative E3 ubiquitin ligase complex and a homolog of CONSTANS. Proc Natl Acad Sci USA. 2019;116:17556–17562. doi:10.1073/pnas.1821689116. 31405963 PMC6717296

[8] Tokutsu R, Fujimura-Kamada K, Matsuo T, Yamasaki T, Minagawa J. The CONSTANS flowering complex controls the protective response of photosynthesis in the green alga Chlamydomonas. Nat Commun. 2019;10:4099. doi:10.1038/s41467-019-11989-x. 31506429 PMC6736836

[9] Sanchez-Barcelo EJ, Mediavilla MD, Vriend J, Reiter RJ. Constitutive photomorphogenesis protein 1 (COP1) and COP9 signalosome, evolutionarily conserved photomorphogenic proteins as possible targets of melatonin. J Pineal Res. 2016;61:41–51. doi:10.1111/jpi.12340. 27121162

[10] Ren X, Chen N, Chen Y, Liu W, Hu Y. TRB3 stimulates SIRT1 degradation and induces insulin resistance by lipotoxicity via COP1. Exp Cell Res. 2019;382:111428. doi:10.1016/j.yexcr.2019.05.009. 31125554

[11] Dornan D, Wertz I, Shimizu H, Arnott D, Frantz GD, Dowd P, O’ Rourke K, Koeppen H, Dixit VM. The ubiquitin ligase COP1 is a critical negative regulator of p53. Nature. 2004;429:86–92. doi:10.1038/nature02514. 15103385

[12] Wertz IE, O’Rourke KM, Zhang Z, Dornan D, Arnott D, Deshaies RJ, Dixit VM. Human de-etiolated-1 regulates c-Jun by assembling a CUL4A ubiquitin ligase. Science. 2004;303:1371–1374. doi:10.1126/science.1093549. 14739464

[13] Yi C, Deng XW. COP1-from plant photomorphogenesis to mammalian tumorigenesis. Trends Cell Biol. 2005;15:618–625. 16198569 10.1016/j.tcb.2005.09.007

[14] Marine J-C. Spotlight on the role of COP1 in tumorigenesis. Nat Rev Cancer. 2012;12:455–464. doi:10.1038/nrc3271. 22673153

[15] Choi HH, Lee M-H. CSN6-COP1 axis in cancer. Aging. 2015;7:461–462. doi:10.18632/aging.100778. 26186957 PMC4543032

[16] Newton K, Dugger DL, Sengupta-Ghosh A, Ferrando RE, Chu F, Tao J, Lam W, Haller S, Chan S, Sa S, et al. Ubiquitin ligase COP1 coordinates transcriptional programs that control cell type specification in the developing mouse brain. Proc Natl Acad Sci USA. 2018;115:11244–11249. doi:10.1073/pnas.1805033115. 30322923 PMC6217379

[17] Jung JH, Seo P, Park CM. The E3 ubiquitin ligase HOS1 regulates Arabidopsis flowering by mediating CONSTANS degradation under cold stress. J Biol Chem. 2012;287:43277–43287. doi:10.1074/jbc.M112.394338. 23135282 PMC3527915

[18] Yin R, Arongaus AB, Binkert M, Ulm R. Two distinct domains of the UVR8 photoreceptor interact with COP1 to initiate UV-B signaling in Arabidopsis. Plant Cell. 2015;27:202–213. doi:10.1105/tpc.114.133868. 25627067 PMC4330580

[19] Jeong R-D, Chandra-Shekara A, Barman SR, Navarre D, Klessig DF, Kachroo A, Kachroo P. Cryptochrome 2 and phototropin 2 regulate resistance protein-mediated viral defense by negatively regulating an E3 ubiquitin ligase. Proc Natl Acad Sci USA. 2010;107:13538–13543. doi:10.1073/pnas.1004529107. 20624951 PMC2922132

[20] Chico J-M, Fernández-Barbero G, Chini A, Fernández-Calvo P, Díez-Díaz M, Solano R. Repression of jasmonate-dependent defenses by shade involves differential regulation of protein stability of MYC transcription factors and their JAZ repressors in Arabidopsis. Plant Cell. 2014;26:1967–1980. doi:10.1105/tpc.114.125047. 24824488 PMC4079362

[21] Kim JY, Jang IC, Seo HS. COP1 controls abiotic stress responses by modulating AtSIZ1 function through its E3 ubiquitin ligase activity. Front Plant Sci. 2016;7:1182. doi:10.3389/fpls.2016.01182. 27536318 PMC4971112

[22] Moazzam-Jazi M, Ghasemi S, Seyedi SM, Niknam V. COP1 plays a prominent role in drought stress tolerance in Arabidopsis and Pea. Plant Physiol Biochem. 2018;130:678–691. doi:10.1016/j.plaphy.2018.08.015. 30139551

[23] Yu Y, Wang J, Shi H, Gu J, Dong J, Deng XW, Huang R. Salt stress and ethylene antagonistically regulate nucleocytoplasmic partitioning of COP1 to control seed germination. Plant Physiol. 2016;170:2340–2350. doi:10.1104/pp.15.01724. 26850275 PMC4825130

[24] Qiu ZB, Wang YF, Zhu AJ, Peng FL, Wang LS. Exogenous sucrose can enhance tolerance of *Arabidopsis thaliana* seedlings to salt stress. Biol Plant. 2014;58:611–617. doi:10.1007/s10535-014-0444-3.

[25] Štefanić PP, Koffler T, Adler G, and Bar-Zvi D. Chloroplasts of salt-grown Arabidopsis seedlings are impaired in structure, genome copy number and transcript levels. PLOS One. 2013;8(12):e82548. 10.1371/journal.pone.0082548 PMC3855474 24340039

[26] Rankenberg T, Geldhof B, van Veen H, Holsteens K, Van de Poel B, Sasidharan R. Age-dependent abiotic stress resilience in plants. Trends Plant Sci. 2021;26(7):692–705. doi:10.1016/j.tplants.2020.12.016. 33509699

[27] Smeekens S, Ma J, Hanson J, Rolland F. Sugar signals and molecular networks controlling plant growth. Curr Opin Plant Biol. 2010;13(3):274–279. doi:10.1016/j.pbi.2009.12.002. 20056477

[28] Wind J, Smeekens S, Hanson J. Sucrose: metabolite and signaling molecule. Phytochemistry. 2010;71(14–15):1610–1614. doi:10.1016/j.phytochem.2010.07.007. 20696445

[29] Ruan YL. Sucrose metabolism: gateway to diverse carbon use and sugar signaling. Annu Rev Plant Biol. 2014;65:33–67. doi:10.1146/annurev-arplant-050213-040251. 24579990

[30] Gong X, Liua M, Zhang L, Ruan Y, Ding R, Ji Y, Zhang N, Zhang S, Farmer J, Wang C. Arabidopsis AtSUC2 and AtSUC4, encoding sucrose transporters, are required for abiotic stress tolerance in an ABA-dependent pathway. Physiol Plant. 2015;153:119–136. doi:10.1111/ppl.12225. 24814155

